# Significance of Parkinson Family Genes in the Prognosis and Treatment Outcome Prediction for Lung Adenocarcinoma

**DOI:** 10.3389/fmolb.2021.735263

**Published:** 2021-09-20

**Authors:** Yanqi Li, Xiao Lu, Jiao Zhang, Quanxing Liu, Dong Zhou, Xufeng Deng, Yuan Qiu, Qian Chen, Manyuan Li, Guixue Yang, Hong Zheng, Jigang Dai

**Affiliations:** ^1^ Department of Thoracic Surgery, Xinqiao Hospital, Army Medical University (Third Military Medical University), Chongqing, China; ^2^ Department of General Surgery, Xinqiao Hospital, Army Medical University (Third Military Medical University), Chongqing, China; ^3^ Cancer Center of Daping Hospital, Third Military Medical University (Army Medical University), Chongqing, China

**Keywords:** Parkinson gene family, LUAD, prognosis, tumour mutation burden, neoantigen, immunotherapy

## Abstract

Epidemiological investigations have shown that patients with Parkinson’s disease (PD) have a lower probability of developing lung cancer. Subsequent research revealed that PD and lung cancer share specific genetic alterations. Therefore, the utilisation of PD biomarkers and therapeutic targets may improve lung adenocarcinoma (LUAD) diagnosis and treatment. We aimed to identify a gene-based signature from 25 Parkinson family genes for LUAD prognosis and treatment choice. We analysed Parkinson family gene expression and protein levels in LUAD, utilising multiple databases. Least absolute shrinkage and selection operator (LASSO) regression was used to construct a prognostic model based on the TCGA-LUAD cohort. We validated the model in external GEO cohorts. Immune cell infiltration was compared between risk groups, and GEO data was used to explore the model’s predictive ability for LUAD treatment response. Nearly all Parkinson family genes exhibited significant differential expression between LUAD and normal tissues. LASSO regression confirmed that our seven Parkinson family gene-based signature had excellent prognostic performance for LUAD, as validated in three GEO cohorts. The high-risk group was clearly associated with low tumour immune cell infiltration, suggesting that immunotherapy may not be an optimal treatment choice. This is the first Parkinson family gene-based model for the prediction of LUAD prognosis and treatment outcome. The association of these genes with poor prognosis and low immune infiltration requires further investigation.

## Introduction

Parkinson’s disease (PD) is the most common neurodegenerative motor disorder. It develops as a result of the premature death of dopamine-containing neurons in a part of the midbrain called the substantia nigra. This leads to a loss of dopaminergic neurons within the substantia nigra pars compacta, depletion of dopamine in the striatum, and the presence of Lewy bodies (Jankovic, 2008). In contrast to the excessive neuronal cell death observed in PD, cancer develops from unrestricted cell proliferation and resistance to cell death (Filippou and Outeiro, 2021). Interestingly, with the development of a more comprehensive understanding of both diseases, an intimate link between PD and lung cancer has been gradually revealed. Most epidemiological studies and meta-analyses have reported a lower incidence of lung cancer in PD patients compared to that in the general population (Catalá-López et al., 2014; Ong et al., 2014; Peretz et al., 2016), highlighting a significant overlap between genes upregulated in PD and downregulated in lung cancer or vice versa (Ibáñez et al., 2014). The intriguing overlap of genes implicated in these two completely different diseases suggests that studying these genes may help improve lung cancer diagnosis, prognosis, and treatment.

Although there is a limited number of studies on Parkinson family genes in cancer, insightful findings have been reported in recent years. Mitophagy, a selective form of autophagy, is the major pathway for the degradation of dysfunctional or superfluous mitochondria in eukaryotic cells (Georgakopoulos et al., 2017), playing a central role in mitochondrial quality control and protection against damaged mitochondria (Tatsuta and Langer, 2008; Youle and Narendra, 2011). The Parkinson family genes PARK2 (PRKN) and PARK6 (PINK1) are considered the main regulators of mitophagy (Yan et al., 2020; Xie et al., 2021). The dysregulation of PRKN- and PINK1-mediated mitophagy has thus been suggested as one of the possible mechanisms underlying the pathogenesis of PD (Lin and Beal, 2006). This notion has been preliminarily validated in mouse models (Lu et al., 2014). Interestingly, mitophagy also has a significant impact on the occurrence and development of tumours. A recent study of hepatocellular carcinoma (HCC) suggested that mitophagy triggered by the accumulation of PINK1 and PRKN translocation can promote the apoptosis of HCC cells, suppressing the growth of patient-derived tumour xenografts (Chen et al., 2019). Further, mitophagy can inhibit the growth of pancreatic tumours by attenuating mitochondrial iron accumulation, inflammasome activation, and other processes, with PINK1 and PRKN depletion confirmed to promote KRAS-driven pancreatic tumourigenesis in mouse models (Kang et al., 2019). PARK18 (EIF4G1), an important part of the EIF4F complex, is required for cap-dependent mRNA translation (Jaiswal et al., 2018). Inevitably, EIF4G1 is involved in various cancer-related processes, such as the activation of the mTOR signalling pathway and hypoxia-inducible factor-1α (HIF-1α)-related processes (Glück et al., 2018; Lu et al., 2021). Other family members such as PARK22 (CHCHD2), which plays an important role in the switch between catabolism and anabolism (Zacksenhaus et al., 2017), PARK7 (DJ-1), which is involved in ferroptosis (Cao et al., 2020), ubiquitination-related regulatory genes PARK15 (FBXO7) and PARK5 (UCHL1) (Goto et al., 2015; Teixeira et al., 2016; Liu et al., 2020), as well as PARK10 (USP24), which is related to cancer-associated acetylation (Wang et al., 2017). In general, the significance of Parkinson family genes in cancer remains to be further explored and could be of major relevance for our understanding of cancer progression.

We have focused on exploring the role of Parkinson family genes in the occurrence and development of cancer. Our previous studies showed that PARK6 (PINK1) can promote migration and proliferation of lung cancer cells by regulating autophagy (Lu et al., 2020), and PARK7 (DJ-1) is necessary for the transcription of HIF-1α and survival of colorectal cancer cells (Lin et al., 2018; Zheng et al., 2018). However, there has been no relevant research on the overall prognostic significance of Parkinson family genes in cancer. Herein, we provide a preliminary analysis of Parkinson family genes in the prognosis and treatment of lung adenocarcinoma (LUAD). The current findings will help us further understand the role of Parkinson family genes in cancer, aid in LUAD prognosis and treatment, and indicate possible directions for future research on this gene family.

## Materials and Methods

### Differential Gene Expression Analysis

We downloaded the normalised gene expression data of cancer and normal tissues from the Cancer Genome Atlas (TCGA) and Genotype-Tissue Expression Project (GTEx) databases in UCSC Xena (http://xena.ucsc.edu/). Data was graphically displayed using the ggplot2 R package. For differences in protein expression between cancer and normal tissues, we used the clinical proteomic tumor analysis consortium (CPTAC) analysis tool of the UALCAN database (http://ualcan.path.uab.edu/index.html), and no LUAD protein expression data were found for PARK6 (PINK1), PARK10 (ELAVL4), and PARK16 (SLC41A1). The expression of Parkinson family genes at different stages of LUAD was explored using the GEPIA2 database (http://gepia2.cancer-pku.cn/#index).

### Survival Analysis

We obtained and downloaded the gene expression and detailed pathological data of 436 LUAD patients and 425 LUSC patients (primary solid tumour samples with detailed prognostic information and a survival time of up to 15 years were included) from the SangerBox database (http://www.sangerbox.com/) to analyse the impact of Parkinson family genes on cancer prognosis. Data was graphically displayed with the help of the survival R package. The forest plot was constructed using GraphPad Prism 8.

### Prognostic Model Construction and Verification

Based on the expression and prognostic value of 25 Parkinson family genes in 436 LUAD and 425 LUSC patient samples, we used the survival R package to construct a Least absolute shrinkage and selection operator (LASSO) regression model. Risk factor analysis was performed using the Hiplot online analysis platform (https://hiplot.com.cn/). A prognostic nomogram model for the 436 LUAD patients was constructed using the rms R package, and the bootstrap method was applied to assess consistency. To validate the model in external data sets, we selected three Gene Expression Omnibus (GEO) chips (GSE37745, GSE31210, and GSE30219) of LUAD patients with detailed prognostic information. The receiver operating characteristic (ROC) curve and time-dependent ROC curve of the working characteristics of subjects were established using R in order to evaluate the survival prediction accuracy of the seven-gene signature for the three chips.

### Bioinformatics Analysis

Univariate and multivariate analyses were conducted using the IBM SPSS Statistics 19. The tumour mutation burden (TMB) and neoantigen (NEO) data of 436 LUAD patients were obtained from the Cancer Imaging Archive (TCIA) database (https://tcia.at/home) (Van Allen et al., 2015; Hugo et al., 2016), and both data were available only for 363 patients. We explored mutations of the seven genes screened via LASSO regression (TCGA, PanCancer Atlas) using the cBioportal database (https://www.cbioportal.org/). Employing the HitPredict database (http://www.hitpredict.org/) (Patil and Nakamura, 2005; Patil et al., 2011; López et al., 2015), we searched for interaction partners of the seven genes. The protein network interaction map was constructed using Cytoscape_v3.7.1. GO and KEGG pathway enrichment analysis of the seven genes and Gene Set Enrichment Analysis (GSEA) analysis of high- and low-risk LUAD patients were carried out using the OmicShare 6.2.1 online tool.

### Immune Cell Infiltration Analysis

In the TIMER database (http://cistrome.dfci.harvard.edu/TIMER/), we explored the impact of mutations in the seven genes on the degree of infiltration for six immune cell types within the tumour microenvironment (TME). Based on the ESTIMATE database (https://bioinformatics.mdanderson.org/estimate/), we further analysed the immune scores of 436 LUAD patients in different risk groups. Next, we used seven analysis methods (TIMER, CIBERSORT, CIBERSORT-ABS, QUANTISEQ, MCPCOUNTER, XCELL, and EPIC) to determine the immune cell infiltration status within the TME of patients in the TIMER2 database (http://timer.cistrome.org/). In addition, we explored differences between the high- and low-risk groups in the steps of the cancer immunity cycle for these patients using the TIP database (http://biocc.hrbmu.edu.cn/TIP/) (Xu et al., 2018).

### Analysis of the Efficacy and Response to Immunotherapy and Targeted Therapy

Gene expression and detailed pathological data of anti-PD-1-treated LUAD patients were obtained from the GSE135222 dataset. The correlation between risk scores and various immunosuppressive molecules was graphically displayed utilising the online analysis tool Hiplot. R packages were used to analyse the correlation between risk score and multiple treatment targets.

## Results

### Expression of Parkinson Family Genes in LUAD

**FIGURE 1:**
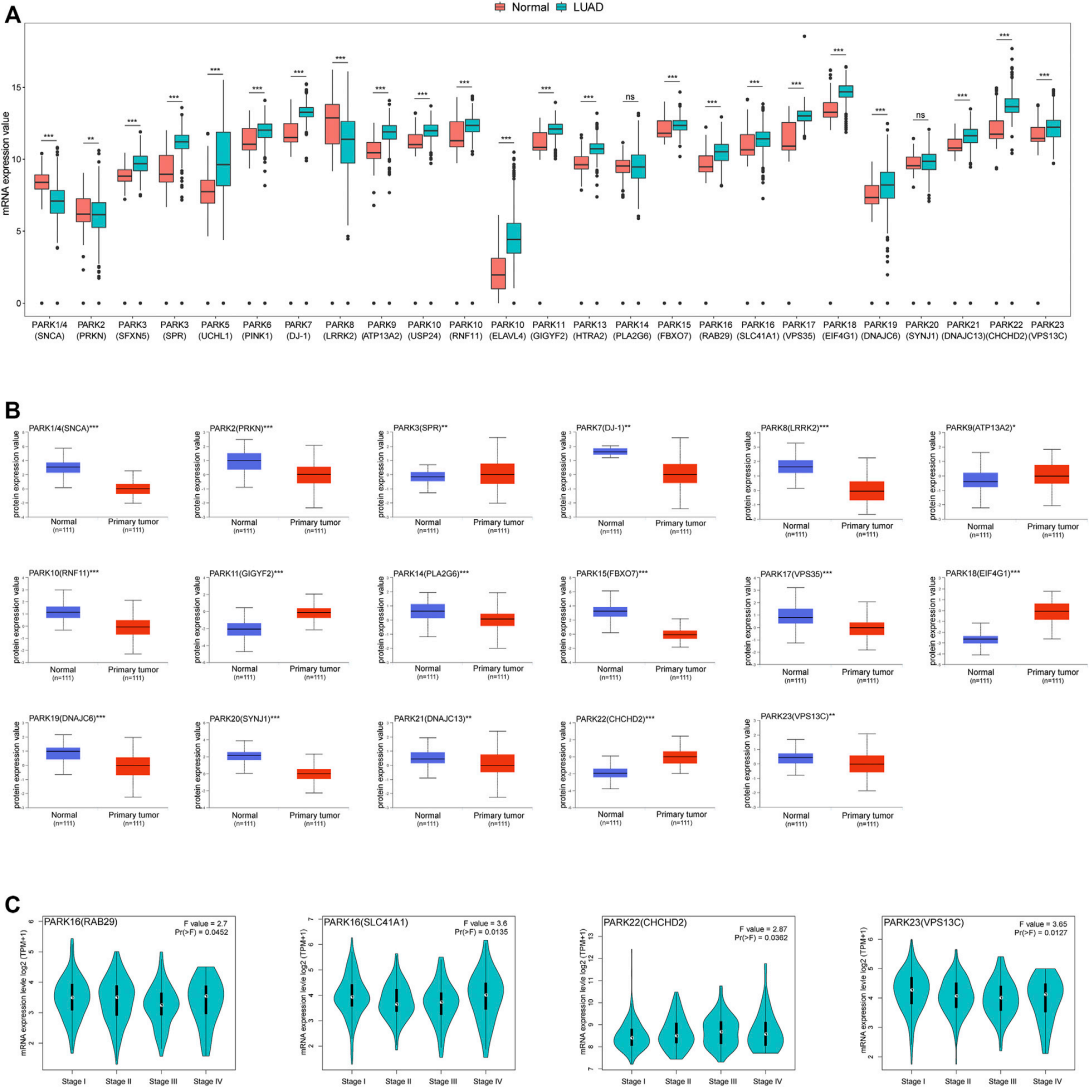
Expression of Parkinson family genes in LUAD. **(A)** Differences in mRNA expression of 25 Parkinson family genes in LUAD and normal tissues from TCGA and GTEx databases (normal = 397, LUAD = 513). **(B)** Differences in the protein expression levels of 17 Parkinson family genes between LUAD and normal tissues in the UALCAN database. **(C)** Expression differences for four Parkinson family genes at different disease stages in the GEPIA2 database. *, **, and *** represent *p* < 0.05, *p* < 0.01, and *p* < 0.001, respectively.

In order to explore the expression of Parkinson family genes in LUAD, we obtained the normalised cancer and normal tissue gene expression data of LUAD patient samples from the TCGA and GTEx databases in UCSC Xena. All Parkinson family genes exhibited significant differential expression, with the exception of PARK14 (PLA2G6) and PARK20 (SYNJ1) (Figure 1A). Similar results were obtained for LUSC (Supplementary Figure S1). Furthermore, using the UALCAN database, we analysed the protein expression of Parkinson family genes between LUAD and normal tissue. Seventeen genes exhibited significant differences in expression at the protein level (Figure 1B). Finally, we explored the expression of Parkinson family genes at different TNM stages in the GEPIA2 database and found that four genes had significant expression differences during the progression of LUAD (Figure 1C). In summary, all Parkinson family genes exhibited significant differential expression in LUAD, suggestive of their involvement in the occurrence and development of lung cancer.

### Prognostic Value of Parkinson Family Genes in LUAD

**FIGURE 2:**
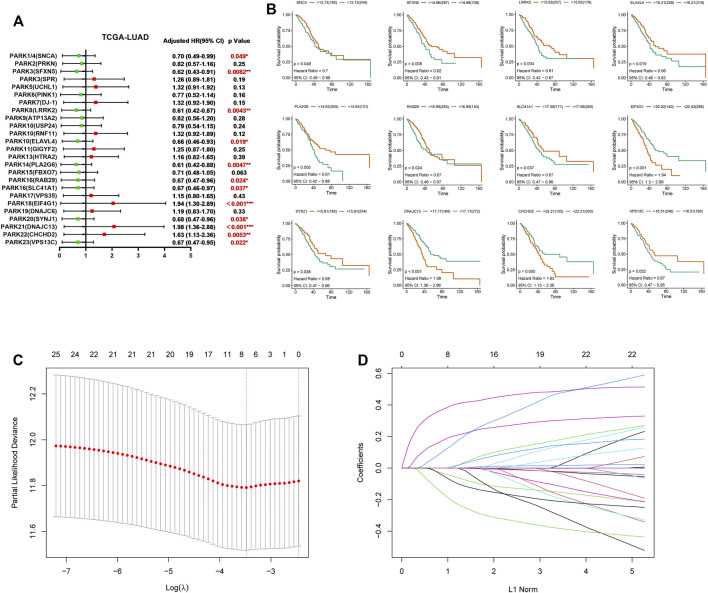
The clinical significance of Parkinson family genes in the prognosis of LUAD and the LASSO regression model. **(A)** LUAD prognostic forest plot of 25 Parkinson family genes from the TCGA database. **(B)** 12 Parkinson family genes with significant differences based on the prognosis of 436 LUAD patients. **(C,D)** LASSO regression prognostic model for the 436 LUAD patients.

Next, we selected 436 LUAD patients from TCGA to investigate the impact of Parkinson family genes on the prognosis of LUAD. We found that 12 genes had significant differences based on LUAD prognosis (Figures 2A,B). Similarly, 11 genes had a significant impact on the prognosis of LUSC patients (Supplementary Figures S2A,B). Moreover, we performed GSEA analysis to explore the influence of deregulation of these genes on 50 hallmark gene sets. Results showed increased expression for these genes associated with poor prognosis can obviously activate a variety of cancer-related pathways (WNT, P53 and NOTCH pathway for LUAD; MYC, G2M checkpoint and Oxidative phosphorylation for LUSC). Interesting, whether in LUAD or LUSC, the impact of the increase of protective prognostic genes and risk genes on the 50 hallmark gene sets were obviously different and even opposed to each other, this finding may provide some help for follow-up in-depth study (Supplementary Figure S3, S4).

However, not all differentially expressed Parkinson genes (in Figure 1A) accurately predicted overall survival in LUAD patients. Therefore, we sought to create a gene signature from 25 Parkinson family genes that had the most pronounced impact on LUAD prognosis. To this end, we constructed a LASSO regression model based on the expression and prognosis data of 436 LUAD patients. We obtained a seven-gene prognostic signature (Figures 2C,D). The complete names of the seven genes and their main functions are listed in Table 1. Meanwhile, we further analyzed the correlation between the 7 genes and genes in the non-small cell lung cancer pathway (map05223) from KEGG database. The results showed that the 7 genes were related to multiple cancer progression marker genes respectively, this result suggest these genes were involved in the development of lung adenocarcinoma via different mechanisms (Supplementary Figure S5). We also constructed a LASSO regression model based on the data of LUSC patients. However, limited by data quality, the results indicated that a combination of two genes had the best performance (Supplementary Figures S2C,D). Therefore, we focused on the seven-gene signature for the prognosis and treatment outcome prediction in LUAD.

**TABLE 1:** LASSO regression result of Parkinson’s disease gene family in TCGA-LUAD dataset.

Gene symbol	Full name	Function
PARK3(SFXN5)	sideroflexin-5	mitochondrial amino-acid transporter
PARK14(PLA2G6)	calcium-independent phospholipase A2	involved in cellular membrane homeostasis, mitochondrial integrity and signal transduction
PARK16(RAB29)	ras-related protein Rab-7L1	Involved in vesicle trafficking
PARK16(SLC41A1)	solute carrier family 41 member 1	a predominant Mg2+ efflux system at the plasma membrane
PARK18(EIF4G1)	eukaryotic translation initiation factor 4 gamma 1	component of the protein complex eIF4F
PARK21(DNAJC13)	dnaJ homolog subfamily C member 13	involved in membrane trafficking through early endosomes
PARK22(CHCHD2)	coiled-coil-helix-coiled-coil-helix domain-containing protein 2	transcription factor

### Our Seven-Gene Signature can be Used as an Independent Prognostic Indicator for LUAD

**FIGURE 3:**
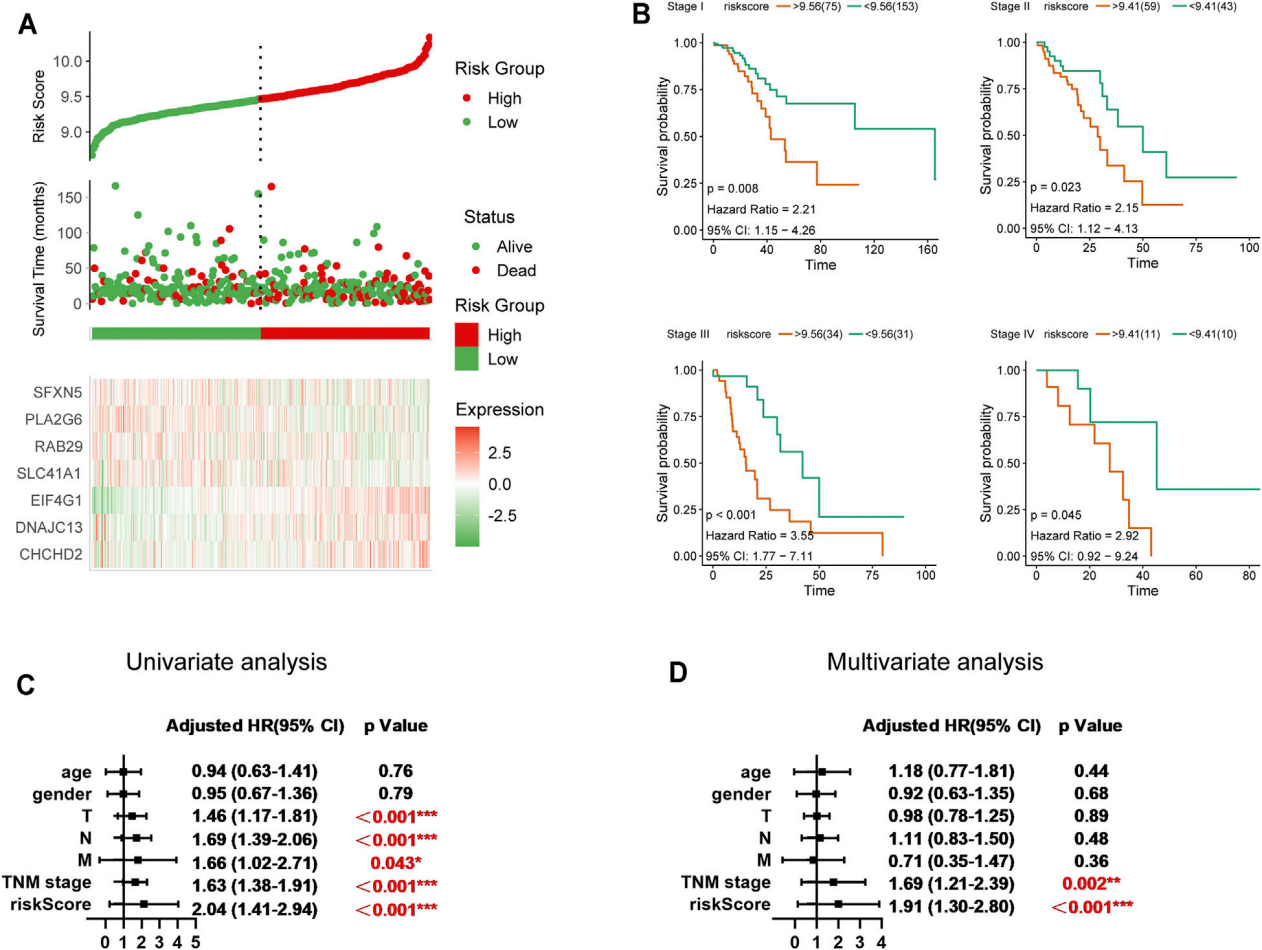
Significance of the seven-gene prognostic model in LUAD. **(A)** Risk factor analysis for the 436 LUAD patients (cut-off value = 9.48). **(B)** Prognostic differences between high- and low-risk groups in different TNM stages in LUAD. **(C,D)** Univariate and multivariate analysis of the seven-gene prognostic risk model.

To better assess the prognostic value of our seven-gene signature in LUAD, we first conducted risk factor analysis based on the genes. The occurrence of death was significantly correlated with a higher risk factor score (Figure 3A). Furthermore, we divided 436 patients into high- and low-risk groups based on risk factor score and analysed prognosis in the two groups. The high-risk group exhibited a significantly poorer prognosis among patients with different TNM stages (Figure 3B). Univariate and multivariate analyses also indicated that the signature-based risk factor score was a superior prognostic indicator compared to TNM staging (Figures 3C,D). Taken together, the seven-gene signature of Parkinson family genes can be used as an independent prognostic marker for LUAD.

### Validation of the Prognostic Value of Our Seven-Gene Signature for LUAD

**FIGURE 4:**
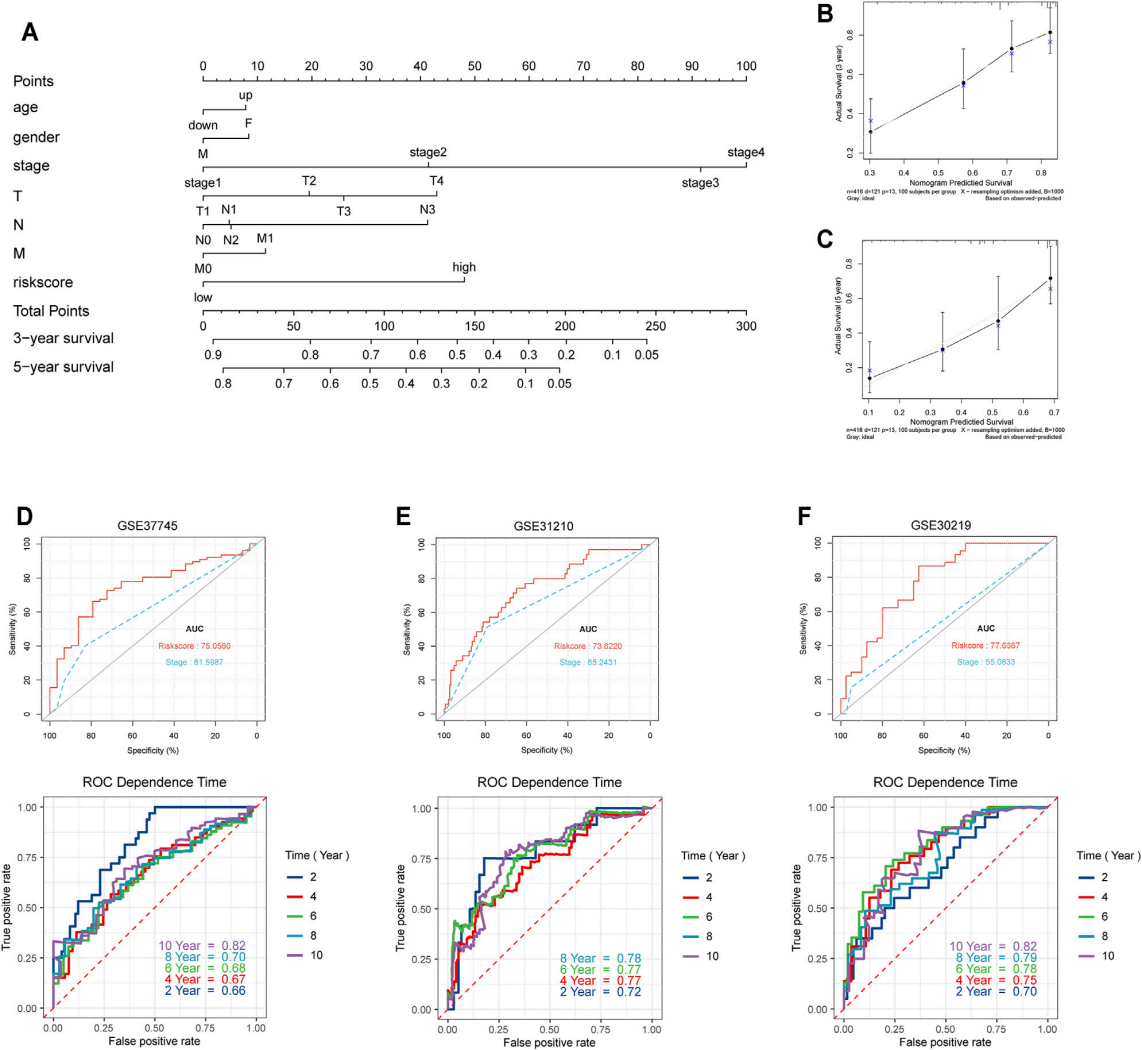
Validation of the seven-gene prognostic risk model. **(A)** The prognostic nomogram for the seven-gene risk model based on the TCGA database. **(B,C)** Bootstrap analysis for determining the 3-years and 5-years survival rates based on the prognostic model. **(D–F)** Validation of the prognostic model in external datasets (GSE37745, GSE31210, and GSE30219).

In order to validate and further assess the prognostic value of our seven-gene signature for LUAD, we constructed a nomogram based on data from the TCGA database in order to predict patient survival probability by weighing age, gender, stage, T, N, M, and the signature-based risk score (Figure 4A). Further, we applied the bootstrap method to evaluate the nomogram’s predictive performance. The calibration curves indicated that the nomogram-predicted probability matched the actual 3- and 5-years survival (Figures 4B,C). Subsequently, we selected three GEO datasets (GSE37745, GSE31210, and GSE30219) with detailed prognostic information for external testing (Table 2). The seven-gene signature risk scores suggested a significantly better diagnostic performance than TNM staging based on the ROC curve, also showing excellent prognostic performance at different survival time points in the time-dependent ROC curve (Figures 4D–F). Taken together, risk evaluation based on the seven-gene signature has important clinical significance in the diagnosis and treatment of LUAD.

**TABLE 2:** GEO dataset for the prognostic value of the 7 genes in LUAD.

Features	GSE37745	GSE31210	GSE30219
Platforms	GPL570	GPL570	GPL570
Samples			
adeno	106	226	85
else	90	20	222
Age			
<60	64	105	118
≥60	132	136	174
Gender			
female	89	130	43
male	107	116	250
Stage			
I	130	168	170
II	35	58	59
III	27	0	55
IV	4	0	8

### Functional Enrichment Analysis of the Seven Signature Genes

**FIGURE 5:**
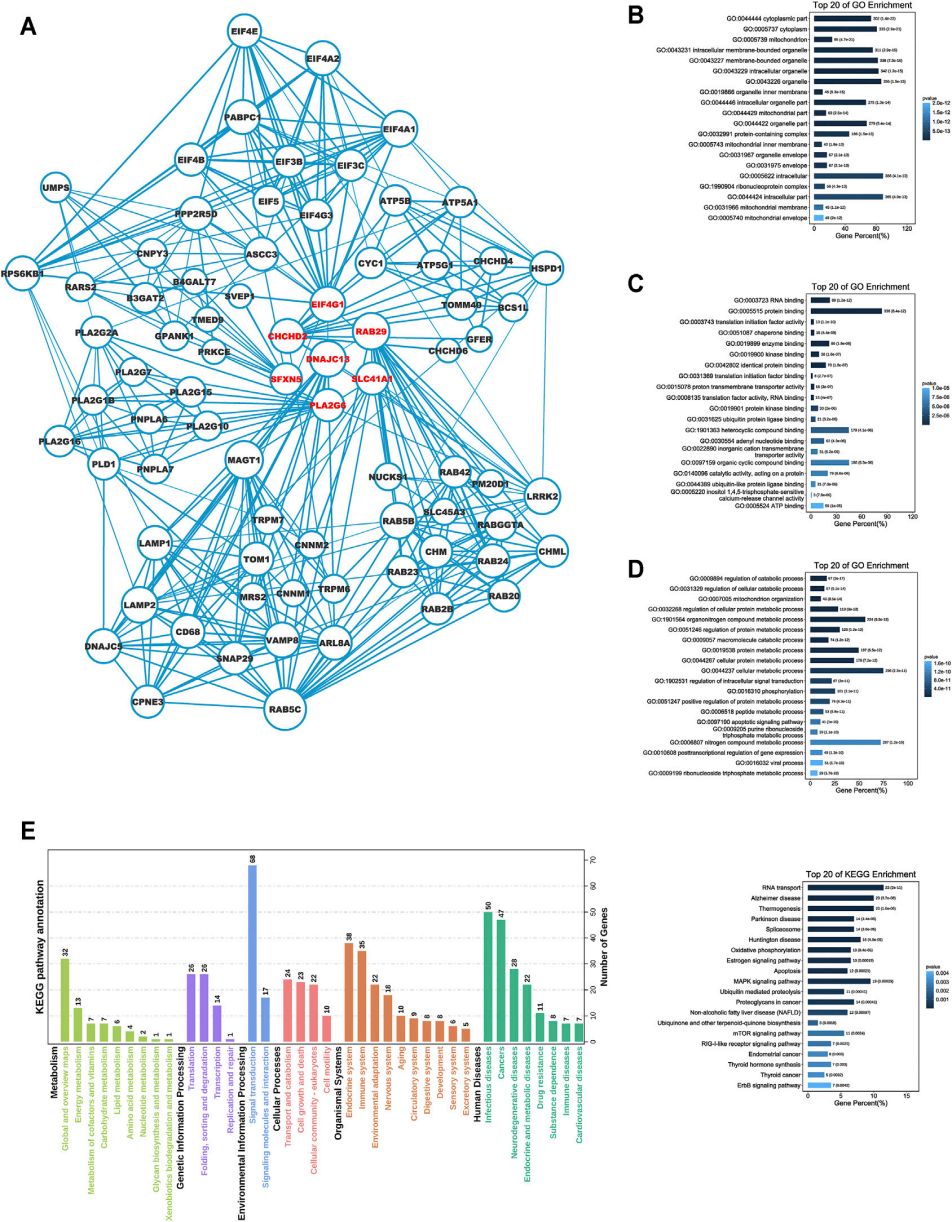
Functional enrichment analysis for the seven Parkinson family genes. **(A)** The protein network interaction map for seven Parkinson family genes based on the HitPredict database. **(B–D)** GO enrichment analysis of the seven Parkinson family genes. **(E)** KEGG pathway enrichment analysis of the seven Parkinson family genes.

We explored the possible mechanisms underlying the functional association of signature genes with poor prognosis. Utilising the HitPredict database, we searched for interaction partners of their protein products, which are displayed in the protein interaction network diagram (Figure 5A). We then performed GO and KEGG enrichment analyses for these genes. GO enrichment analysis indicated the genes’ involvement in diverse tumourigenesis- and development-associated signalling, such as metabolism-related pathways, ubiquitination pathways, and RNA transcription-related processes (Figures 5B–D). Furthermore, KEGG pathway enrichment analysis also revealed that immune and cell proliferation-related pathways (RIG-I-like receptor, MAPK, mTOR, and ErBb) were enriched by these genes (Figure 5E).

### Signature Gene Expression Affects the TME in LUAD

**FIGURE 6:**
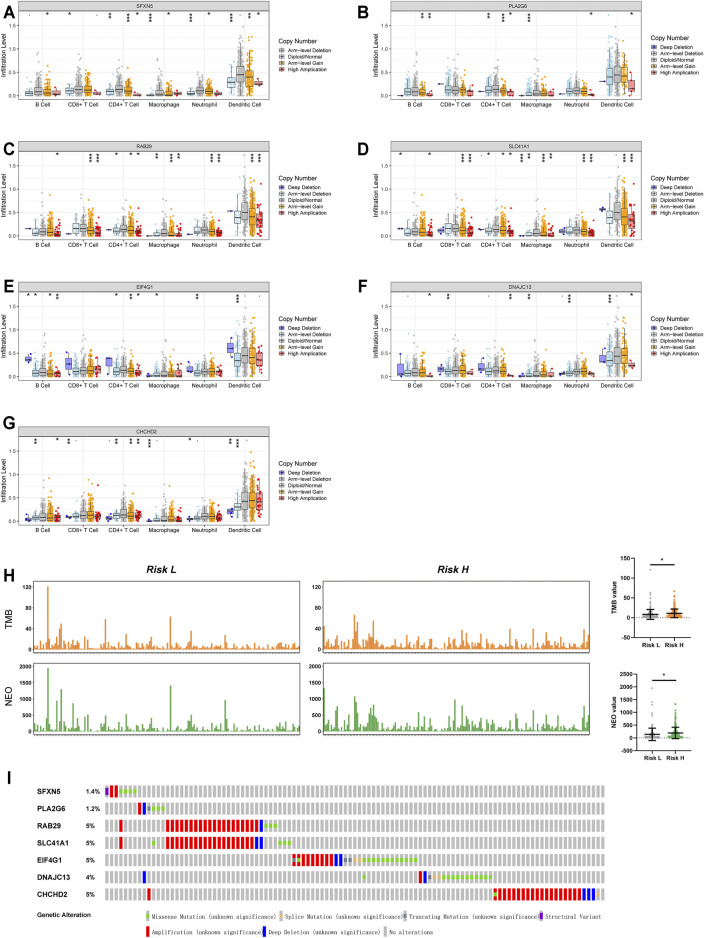
The effect of mutations in the seven Parkinson family genes on immune cell infiltration within the TME. **(A–G)** The effect of mutations in SFXN5, PLA2G6, RAB29, SLC41A1, EIF4G1, DNAJC13, and CHCHD2 on the infiltration of six immune cell types (B cell, CD8+T cell, CD4+T cell, macrophage, neutrophil, dendritic cell) in the TME based on the TIMER database. **(H)** Mutations of TMB and NEO in high- and low-risk groups in the TCGA database. **(I)** Mutation status of the seven Parkinson family genes in LUAD data from TCGA.

In view of the fact that various immune-related processes were also observed among functional enrichment results (Figure 5E), we sought to explore the influence of the seven signature genes on the TME. Employing the TIMER database, we obtained the effect of various single gene mutations on the infiltration of six immune cell types (B cells, CD8+T cells, CD4+T cells, macrophages, neutrophils, and dendritic cells) into the TME. The results indicated that mutations of signature genes decreased the degree of infiltration for almost all six immune cell types (Figures 6A–G). We also explored the link between risk factor score and TMB (or NEO) based on TCIA data. A high-risk score was clearly related to a high TMB and high NEO (Figure 6H). Mutation data from the cBioportal database indicated that mutations in these seven genes were rare among TCGA-LUAD patients (Figure 6I).

**FIGURE 7:**
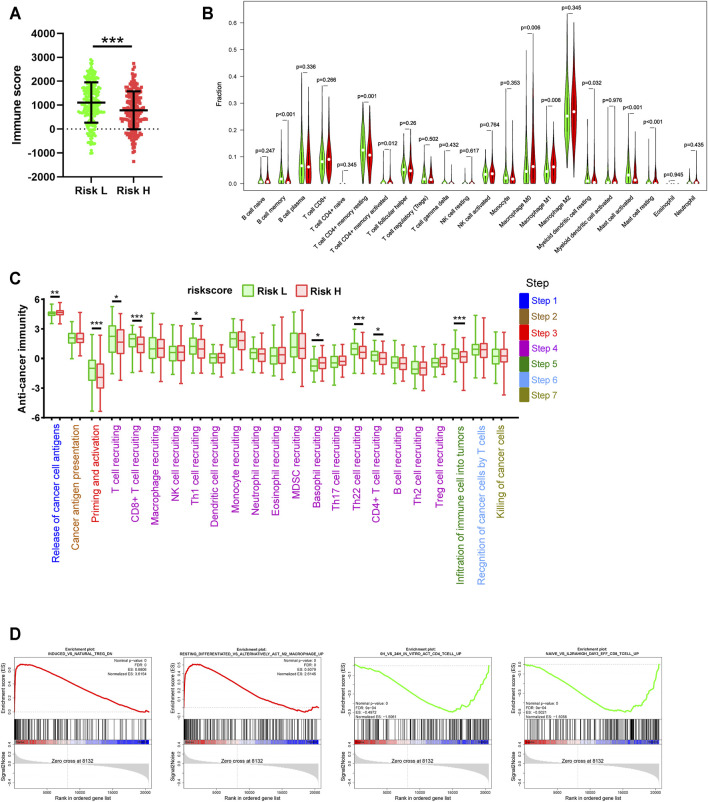
Differences in tumor immune-related processes between high- and low-risk groups. **(A)** Estimation analysis of the immune scores for high- and low-risk groups. **(B)** Differences in the infiltration status of 22 immune cell types between high- and low-risk groups via the CIBERSORT method. **(C)** Differences in the cancer immunity cycle between high- and low-risk groups. **(D)** GSEA analysis for high- and low-risk groups based on TCGA data.

We further explored the effect of the seven-gene signature on the TME. Estimation analysis indicated that the immune score of the high-risk group was significantly lower than that of the low-risk group for the 436 LUAD patients (Figure 7A). We further observed the different infiltration levels of 22 immune cell types between the low- and high-risk groups using CIBERSORT (Figure 7B). Meanwhile, in order to eliminate possible errors caused by different analysis methods, we employed another six methods (Supplementary Figure S6) and summarised the results, revealing the same trend and significant differences (Table 3). However, these results revealed an interesting phenomenon. In the high-risk group, the infiltration of DC cells, B cells, CD4+ T cells, and CD8+ T cells remained lower, while M1 pro-inflammatory macrophages were upregulated, and M2 anti-inflammatory macrophages and Treg cells were downregulated.

**TABLE 3:** The correlation between the risk score and immune cell infiltration level.

	TIMER	CIBERSORT.ABS	QUANTISEQ	MCPCOUNTER	XCELL	EPIC
B_cell	−0.29***	Naïve: −0.06, Memory: −0.23***, Plasma: −0.14**	−0.25***	−0.24***	Naïve: −0.12*, Memory: −0.22***, Plasma: −0.14**	−0.23***
CD4+T_cell	−0.27***	Naïve: −0.0018, Resting_memory: −0.27***, Activatied_memory: 0.11*	0.052	Null	Naïve: −0.25***, Non_regulatory: −0.14**, Central_memory: −0.27***, Effector_memory: −0.21***	−0.075
CD8+T_cell	−0.041	-0.070	−0.057	−0.064	Naive: 0.0055, Centra memory: −0.16***, Effector memory: −0.052	−0.15**
Macrophage	−0.14**	M0: 0.077, M1: 0.027*, M2: −0.17***	M1: 0.080, M2: −0.31***	0.064	M1: 0.020*, M2: −0.26***	−0.15**
Dendritic_cell	−0.12*	Resting: −0.18***, Activated: −0.036	0.12**	−0.26***	Activated: -0.18***, Plasmacytoid: −0.051	Null
Tregs_cell	Null	−0.15**	−0.22***	Null	−0.037	Null
Mast_cell	Null	Resting: 0.14** Activated: −0.31***	Null	Null	-0.17***	Null
Eosinophil	Null	−0.069	Null	Null	−0.29***	Null
Endothelial_cell	Null	Null	Null	−0.20***	−0.16**	−0.18***

*, **, and *** represent p < 0.05, p < 0.01, p < 0.001, respectively.

The cancer immunity cycle reflects a complex interaction network involving the chemokine system, other immunomodulators, and the various immune cell types. Based on TIP data, we found that high risk was associated with the release of cancer cell antigens (Step 1), reduced priming and activation (Step 3), recruitment of multiple immune cells (Step 4), and the infiltration of immune cells into tumours (Step 5) (Figure 7C). It is worth noting that Th1 cells were significantly downregulated in the high-risk group, while Th2 cells were upregulated, although the difference was not statistically significant. This partly explains why the various effector cells of cellular immunity exhibited low infiltration with an opposite trend of change compared to regulatory cells. The detailed underlying mechanism remains to be further explored. Similarly, GSEA analysis showed that signature-based high risk was associated with a significant inhibition of tumour immune-related processes (Figure 7D). Exploring the mechanism underlying the increase in M2 cells and Treg cells in the high-risk group may help explain the low infiltration of effector cells.

### Utility of the Seven-Gene Signature for the Prediction of Immunotherapy and Targeted Therapy Response in LUAD

**FIGURE 8:**
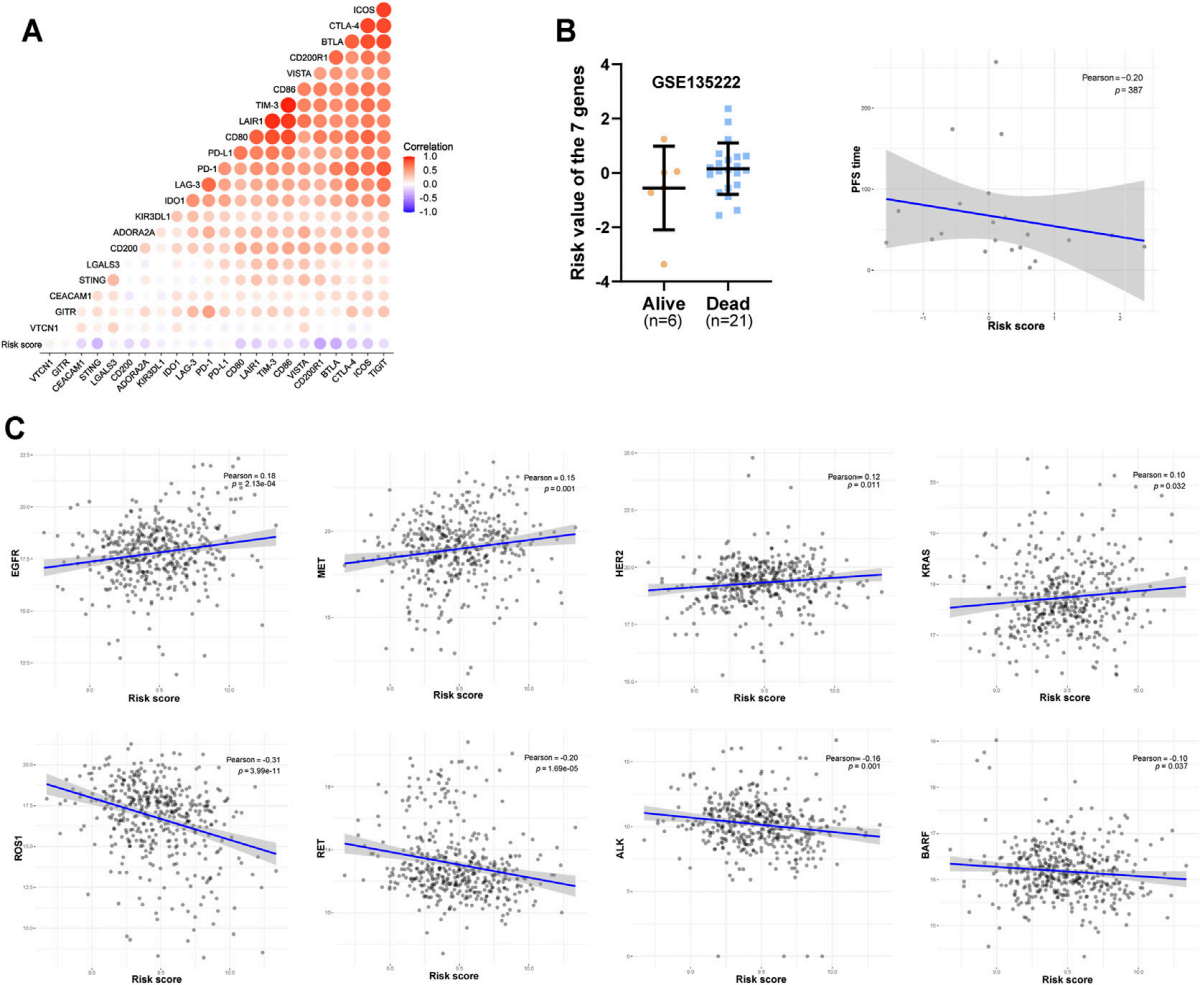
Predictive value of the seven-gene signature for LUAD immunotherapy and targeted therapy outcomes. **(A)** Heat map of the correlation between risk score and the expression of multiple immunosuppressive regulatory molecules. **(B)** The correlation between the risk score and the efficacy of PD-1 immunotherapy based on the treatment data of GSE135222. **(C)** Correlation between risk score and treatment outcome of LUAD patients.

Finally, we sought to explore the utility of our seven-gene signature as a predictor of immunotherapy and targeted therapy response in LUAD. A high-risk score was significantly negatively correlated with a variety of immunosuppressive molecules (Figure 8A). Furthermore, we explored the correlation between risk score and the efficacy of PD-L1 immunotherapy in LUAD. Data from GSE135222 indicated that after PD-1 immune checkpoint inhibitor treatment, the risk score of dying patients was higher than that of surviving patients, and there was a negative correlation between the survival time for surviving patients and the risk score (Figure 8B). Limited by the sample size, the results were not statistically significant, and we did not find other available immunotherapy cohorts with detailed prognostic and gene expression data. Nevertheless, we believe that these results would be useful for treatment selection. We analysed the correlation between risk score and the expression of multiple driver genes for targeted therapy in TCGA patients, observing a clear correlation (Figure 8C). Furthermore, based on the Genomics of Drug Sensitivity in Cancer (GDSC) database, we predicted the impact of 7 genes on the half-inhibitory concentration (IC50) of some common chemotherapeutics (platinum, paclitaxel, etc) and targeted agents for non-small cell lung cancer (NSCLC). Interestingly, the result showed high expression of SLC41A1, RAB29 and PLA2G6 may be positively correlated with resistance of various common chemotherapeutics, whereas high level of CHCHD2, EIF4G1, DNAJC13 and SFXN5 may be involved in resistance of target therapy for drive gene (Supplementary Figure S7). However, we did not find available targeted therapy datasets for validation. In general, our findings indicated that targeted therapy may be more appropriate than immunotherapy for high-risk patients as a precision medicine approach for LUAD.

## Discussion

Lung cancer remains the major contributor to the cancer disease burden worldwide, ranking first in cancer-associated mortality. The biggest reason for this is that lung cancer patients have already entered the middle or late stages of disease upon diagnosis. Advanced disease has a strong tendency for metastasis and relapse. Although progress has been made in diagnosis and treatment strategies over the past decades, patient prognosis remains very poor, with a 5-years survival rate of only about 20% (Siegel et al., 2021). As LUAD is the major histologic subtype of lung cancer, it is necessary to explore more effective and sensitive biomarkers for its prognosis and treatment.

Instead of basing LUAD prognosis on a single gene, we screened 25 Parkinson family genes and established a seven-gene prognostic signature (PARK3 [SFXN5], PARK14 [PLA2G6], PARK16 [RAB29], PARK16 [SLC41A1], PARK18 [EIF4G1], PARK21 [DNAJC13], PARK22 [CHCHD2]) for LUAD. Whether in the TCGA-LUAD cohort or the three GEO cohorts for external verification, the seven-gene signature exhibited excellent performance for LUAD prognosis. Although previous studies have shown that the signature genes were related to a variety of cancers (liver cancer, breast cancer, oral cancer, and others), there have been few studies on the mechanism through which they participate in cancer progression (Li et al., 2017; Khowal et al., 2018; Uddin et al., 2018; Yao et al., 2019). Migration, proliferation, energy metabolism, and autophagy are all considered regulatory targets, but the underlying mechanism remains to be further elucidated (Hosgood et al., 2008; Wei et al., 2015; Jaiswal et al., 2018; Ma et al., 2020; Wang et al., 2021). In addition, HIF-1α, a key regulator of the tumour hypoxic response, may be implicated in the signature genes’ association with poor prognosis. Studies have shown that in non-small cell lung cancer (NSCLC), CHCHD2 and HIF-1α co-localise in both the cytoplasm and the nucleus, with CHCHD2 overexpression significantly promoting that of HIF-1α (Yin et al., 2020). EIF4G1 was also confirmed as involved in HIF-1α overexpression in NSCLC (Glück et al., 2018). Furthermore, studies have suggested that EIF4G1 can also promote NSCLC progression by regulating the expression and phosphorylation of mTOR (Ser 2448) (Lu et al., 2021). In general, the current understanding of the signature genes’ functions in cancer is relatively limited, necessitating further investigation.

As the seven genes belong to the Parkinson family, understanding how they contribute to PD may be conducive for future studies in cancer. Unfortunately, the research on SFXN5, SCL41A1, EIF4G1, and DNAJC13 in PD is limited (Deng et al., 2015; Wang et al., 2015; Puschmann, 2017), and their specific mechanisms in PD pathogenesis remain unclear. Nevertheless, some studies have provided valuable insights. PLA2G6 is believed to induce PD mainly by disturbing lipid metabolism in neurons. When lipid metabolism is dysregulated, the transient interaction between α-synuclein and the synaptic vesicle membrane composed of phospholipids and other lipids was affected, resulting in α-synuclein aggregation and the resultant pathological α-synuclein conversion (Mori et al., 2020). At the same time, oxidative stress and inflammation caused by an imbalance of lipid metabolism also contribute to the occurrence and development of PD (Alecu and Bennett, 2019). In addition, RAB29 is believed to cause PD via lysosomal dysfunction, while CHCHD2 may cause PD by impairing mitochondrial function (Zhou et al., 2019; Mazza et al., 2021). These processes are also of major relevance in tumours, providing an avenue for their further investigation in cancer.

Finally, we analysed the predictive merit of our seven-gene signature for LUAD treatment outcome. We observed that a high risk based on the signature may be related to low immune cell infiltration in LUAD, which was not underpinned by the polarisation of M2 macrophages and Treg cell infiltration. We further found that PD-1 immunotherapy may not be optimal for high-risk patients, as a high risk was related to low immune scores, suggesting targeted therapy as a more suitable option. Recent research has shown that tumour-associated macrophages (TAMs) are critical mediators of the PD-1/PD-L1 axis, and the high infiltration of M2 macrophages is significantly correlated with high PD-L1 expression within the TME (Zhu et al., 2020). Furthermore, SPP1 can promote the polarisation of M2 macrophages, and knockdown of SPP1 significantly suppressed PD-L1 expression on LUAD cells (Zhang et al., 2017). Other specific immunoregulatory mechanisms of signature genes required further investigation. Nevertheless, we consider the current findings to be of considerable value for the treatment of high-risk LUAD patients.

In summary, our study is the first to explore the role of Parkinson family genes in the prognosis and treatment of LUAD. Our research showed that the combination of seven Parkinson family genes may help predict LUAD prognosis as well as the treatment outcome for high-risk patients. Our study provides novel insight into the significance of Parkinson family genes in LUAD. We hope that the current findings will be of value for the clinical diagnosis and treatment of LUAD, and, more importantly, will further the exploration of Parkinson family genes in cancer. However, our results need more support and validation from clinical and animal models, continue to explore the unknown function and specific mechanisms of Parkinson family genes in tumors is a potential direction**.**

## Data Availability

The original contributions presented in the study are included in the article/Supplementary Material, further inquiries can be directed to the corresponding authors.

## References

[B1] Alecu I. Bennett S. A. L. (2019). Dysregulated Lipid Metabolism and its Role in α-Synucleinopathy in Parkinson's Disease. Front. Neurosci. 13, 328. 10.3389/fnins.2019.00328 31031582 PMC6470291

[B2] Cao J. Chen X. Jiang L. Lu B. Yuan M. Zhu D. (2020). DJ-1 Suppresses Ferroptosis through Preserving the Activity of S-Adenosyl Homocysteine Hydrolase. Nat. Commun. 11, 1251. 10.1038/s41467-020-15109-y 32144268 PMC7060199

[B3] Catalá-López F. Suárez-Pinilla M. Suárez-Pinilla P. Valderas J. M. Gómez-Beneyto M. Martinez S. (2014). Inverse and Direct Cancer Comorbidity in People with Central Nervous System Disorders: A Meta-Analysis of Cancer Incidence in 577,013 Participants of 50 Observational Studies. Psychother Psychosom 83, 89–105. 10.1159/000356498 24458030

[B4] Chen Y. Chen H.-N. Wang K. Zhang L. Huang Z. Liu J. (2019). Ketoconazole Exacerbates Mitophagy to Induce Apoptosis by Downregulating Cyclooxygenase-2 in Hepatocellular Carcinoma. J. Hepatol. 70, 66–77. 10.1016/j.jhep.2018.09.022 30287340

[B5] Deng H. Wu Y. Jankovic J. (2015). TheEIF4G1gene and Parkinson's Disease. Acta Neurol. Scand. 132, 73–78. 10.1111/ane.12397 25765080

[B6] Filippou P. S. Outeiro T. F. (2021). Cancer and Parkinson's Disease: Common Targets, Emerging Hopes. Mov Disord. 36, 340–346. 10.1002/mds.28425 33346940

[B7] Georgakopoulos N. D. Wells G. Campanella M. (2017). The Pharmacological Regulation of Cellular Mitophagy. Nat. Chem. Biol. 13, 136–146. 10.1038/nchembio.2287 28103219

[B8] Glück A. A. Orlando E. Leiser D. Poliaková M. Nisa L. Quintin A. (2018). Identification of a MET-eIF4G1 Translational Regulation axis that Controls HIF-1α Levels under Hypoxia. Oncogene 37, 4181–4196. 10.1038/s41388-018-0256-6 29717265

[B9] Goto Y. Zeng L. Yeom C. J. Zhu Y. Morinibu A. Shinomiya K. (2015). UCHL1 Provides Diagnostic and Antimetastatic Strategies Due to its Deubiquitinating Effect on HIF-1α. Nat. Commun. 6, 6153. 10.1038/ncomms7153 25615526 PMC4317501

[B10] Hosgood H. D. Menashe I. Shen M. Yeager M. Yuenger J. Rajaraman P. (2008). Pathway-based Evaluation of 380 Candidate Genes and Lung Cancer Susceptibility Suggests the Importance of the Cell Cycle Pathway. Carcinogenesis 29, 1938–1943. 10.1093/carcin/bgn178 18676680 PMC2722857

[B11] Hugo W. Zaretsky J. M. Sun L. Song C. Moreno B. H. Hu-Lieskovan S. (2016). Genomic and Transcriptomic Features of Response to Anti-PD-1 Therapy in Metastatic Melanoma. Cell 165, 35–44. 10.1016/j.cell.2016.02.065 26997480 PMC4808437

[B12] Ibáñez K. Boullosa C. Tabarés-Seisdedos R. Baudot A. Valencia A. (2014). Molecular Evidence for the Inverse Comorbidity between central Nervous System Disorders and Cancers Detected by Transcriptomic Meta-Analyses. Plos Genet. 10, e1004173. 10.1371/journal.pgen.1004173 24586201 PMC3930576

[B13] Jaiswal P. K. Koul S. Shanmugam P. S. T. Koul H. K. (2018). Eukaryotic Translation Initiation Factor 4 Gamma 1 (eIF4G1) Is Upregulated during Prostate Cancer Progression and Modulates Cell Growth and Metastasis. Sci. Rep. 8, 7459. 10.1038/s41598-018-25798-7 29748619 PMC5945649

[B14] Jankovic J. (2008). Parkinson's Disease: Clinical Features and Diagnosis. J. Neurol. Neurosurg. Psychiatry 79, 368–376. 10.1136/jnnp.2007.131045 18344392

[B15] Kang R. Xie Y. Zeh H. J. Klionsky D. J. Tang D. (2019). Mitochondrial Quality Control Mediated by PINK1 and PRKN: Links to Iron Metabolism and Tumor Immunity. Autophagy 15, 172–173. 10.1080/15548627.2018.1526611 30252570 PMC6287677

[B16] Khowal S. Naqvi S. H. Monga S. Jain S. K. Wajid S. (2018). Assessment of Cellular and Serum Proteome from Tongue Squamous Cell Carcinoma Patient Lacking Addictive Proclivities for Tobacco, Betel Nut, and Alcohol: Case Study. J. Cel. Biochem. 119, 5186–5221. 10.1002/jcb.26554 29236289

[B17] Li M. Li C. Liu W.-X. Liu C. Cui J. Li Q. (2017). Dysfunction of PLA2G6 and CYP2C44-Associated Network Signals Imminent Carcinogenesis from Chronic Inflammation to Hepatocellular Carcinoma. J. Mol. Cel Biol 9, 489–503. 10.1093/jmcb/mjx021 PMC5907842 28655161

[B18] Lin M. T. Beal M. F. (2006). Mitochondrial Dysfunction and Oxidative Stress in Neurodegenerative Diseases. Nature 443, 787–795. 10.1038/nature05292 17051205

[B19] Lin Y. Chen Q. Liu Q.-x. Zhou D. Lu X. Deng X.-f. (2018). High Expression of DJ-1 Promotes Growth and Invasion via the PTEN-AKT Pathway and Predicts a Poor Prognosis in Colorectal Cancer. Cancer Med. 7, 809–819. 10.1002/cam4.1325 29441725 PMC5852339

[B20] Liu S. González-Prieto R. Zhang M. Geurink P. P. Kooij R. Iyengar P. V. (2020). Deubiquitinase Activity Profiling Identifies UCHL1 as a Candidate Oncoprotein that Promotes TGFβ-Induced Breast Cancer Metastasis. Clin. Cancer Res. 26, 1460–1473. 10.1158/1078-0432.CCR-19-1373 31857432 PMC7611208

[B21] López Y. Nakai K. Patil A. (2015). HitPredict Version 4: Comprehensive Reliability Scoring of Physical Protein-Protein Interactions from More Than 100 Species. Database 2015, bav117. 10.1093/database/bav117 26708988 PMC4691340

[B22] Lu W. Karuppagounder S. S. Springer D. A. Allen M. D. Zheng L. Chao B. (2014). Genetic Deficiency of the Mitochondrial Protein PGAM5 Causes a Parkinson's-like Movement Disorder. Nat. Commun. 5, 4930. 10.1038/ncomms5930 25222142 PMC4457367

[B23] Lu X. Liu Q.-X. Zhang J. Zhou D. Yang G.-X. Li M.-Y. (2020). PINK1 Overexpression Promotes Cell Migration and Proliferation via Regulation of Autophagy and Predicts a Poor Prognosis in Lung Cancer Cases. Cancer. Manag. Res. Vol. 12, 7703–7714. 10.2147/CMAR.S262466 PMC7457709 32904694

[B24] Lu Y. Yu S. Wang G. Ma Z. Fu X. Cao Y. (2021). Elevation of EIF4G1 Promotes Non‐small Cell Lung Cancer Progression by Activating mTOR Signalling. J. Cel Mol Med 25, 2994–3005. 10.1111/jcmm.16340 PMC7957198 33523588

[B25] Ma L. Zheng L. H. Zhang D. G. Fan Z. M. (2020). CHCHD2 Decreases Docetaxel Sensitivity in Breast Cancer via Activating MMP2. Eur. Rev. Med. Pharmacol. Sci. 24, 6426–6433. 10.26355/eurrev_202006_21541 32572940

[B26] Mazza M. C. Nguyen V. Beilina A. Karakoleva E. Coyle M. Ding J. (2021). Combined Knockout of Lrrk2 and Rab29 Does Not Result in Behavioral Abnormalities *In Vivo* . J. Parkinson. Dis. 11, 569–584. 10.3233/JPD-202172 PMC9272729 33523017

[B27] Mori A. Imai Y. Hattori N. (2020). Lipids: Key Players that Modulate α-Synuclein Toxicity and Neurodegeneration in Parkinson's Disease. Int. J. Mol. Sci. 21, 3301. 10.3390/ijms21093301 PMC7247581 32392751

[B28] Ong E. L. Goldacre R. Goldacre M. (2014). Differential Risks of Cancer Types in People with Parkinson's Disease: A National Record-Linkage Study. Eur. J. Cancer 50, 2456–2462. 10.1016/j.ejca.2014.06.018 25065294

[B29] Patil A. Nakai K. Nakamura H. (2011). HitPredict: a Database of Quality Assessed Protein-Protein Interactions in Nine Species. Nucleic Acids Res. 39, D744–D749. 10.1093/nar/gkq897 20947562 PMC3013773

[B30] Patil A. Nakamura H. (2005). Filtering High-Throughput Protein-Protein Interaction Data Using a Combination of Genomic Features. Bmc Bioinformatics 6, 100. 10.1186/1471-2105-6-100 15833142 PMC1127019

[B31] Peretz C. Gurel R. Rozani V. Gurevich T. El-Ad B. Tsamir J. (2016). Cancer Incidence Among Parkinson's Disease Patients in a 10-yrs Time-Window Around Disease Onset: A Large-Scale Cohort Study. Parkinsonism Relat. Disord. 28, 68–72. 10.1016/j.parkreldis.2016.04.028 27161827

[B32] Puschmann A. (2017). New Genes Causing Hereditary Parkinson's Disease or Parkinsonism. Curr. Neurol. Neurosci. Rep. 17, 66. 10.1007/s11910-017-0780-8 28733970 PMC5522513

[B33] Siegel R. L. Miller K. D. Fuchs H. E. Jemal A. (2021). Cancer Statistics, 2021. CA A. Cancer J. Clin. 71, 7–33. 10.3322/caac.21654 33433946

[B34] Tatsuta T. Langer T. (2008). Quality Control of Mitochondria: Protection against Neurodegeneration and Ageing. Embo J. 27, 306–314. 10.1038/sj.emboj.7601972 18216873 PMC2234350

[B35] Teixeira F. R. Randle S. J. Patel S. P. Mevissen T. E. T. Zenkeviciute G. Koide T. (2016). Gsk3β and Tomm20 Are Substrates of the SCFFbxo7/PARK15 Ubiquitin Ligase Associated with Parkinson's Disease. Biochem. J. 473, 3563–3580. 10.1042/BCJ20160387 27503909 PMC5260939

[B36] Uddin M. Balaravi Pillai B. Tha K. Ashaie M. Karim M. Chowdhury E. (2018). Carbonate Apatite Nanoparticles-Facilitated Intracellular Delivery of siRNA(s) Targeting Calcium Ion Channels Efficiently Kills Breast Cancer Cells. Toxics 6, 34. 10.3390/toxics6030034 PMC6161028 29949888

[B37] Van Allen E. M. Miao D. Schilling B. Shukla S. A. Blank C. Zimmer L. (2015). Genomic Correlates of Response to CTLA-4 Blockade in Metastatic Melanoma. Science 350, 207–211. 10.1126/science.aad0095 26359337 PMC5054517

[B38] Wang F. Liao Y. Zhang M. Zhu Y. Wang W. Cai H. (2021). N6-methyladenosine Demethyltransferase FTO-Mediated Autophagy in Malignant Development of Oral Squamous Cell Carcinoma. Oncogene 40, 3885–3898. 10.1038/s41388-021-01820-7 33972683

[B39] Wang L. Cheng L. Li N.-N. Yu W.-J. Sun X.-Y. Peng R. (2015). Genetic Analysis ofSLC41A1in Chinese Parkinson's Disease Patients. Am. J. Med. Genet. 168, 706–711. 10.1002/ajmg.b.32365 26308152

[B40] Wang S.-A. Wang Y.-C. Chuang Y.-P. Huang Y.-H. Su W.-C. Chang W.-C. (2017). EGF-mediated Inhibition of Ubiquitin-specific Peptidase 24 Expression Has a Crucial Role in Tumorigenesis. Oncogene 36, 2930–2945. 10.1038/onc.2016.445 27991932 PMC5454318

[B41] Wei Y. Vellanki R. N. Coyaud É. Ignatchenko V. Li L. Krieger J. R. (2015). CHCHD2 Is Coamplified with EGFR in NSCLC and Regulates Mitochondrial Function and Cell Migration. Mol. Cancer Res. 13, 1119–1129. 10.1158/1541-7786.MCR-14-0165-T 25784717

[B42] Xie Y. Liu J. Kang R. Tang D. (2021). Mitophagy in Pancreatic Cancer. Front. Oncol. 11, 616079. 10.3389/fonc.2021.616079 33718171 PMC7953903

[B43] Xu L. Deng C. Pang B. Zhang X. Liu W. Liao G. (2018). TIP: A Web Server for Resolving Tumor Immunophenotype Profiling. Cancer Res. 78, 6575–6580. 10.1158/0008-5472.CAN-18-0689 30154154

[B44] Yan C. Gong L. Chen L. Xu M. Abou-Hamdan H. Tang M. (2020). PHB2 (Prohibitin 2) Promotes PINK1-PRKN/Parkin-dependent Mitophagy by the PARL-PGAM5-PINK1 axis. Autophagy 16, 419–434. 10.1080/15548627.2019.1628520 31177901 PMC6999623

[B45] Yao Y. Su J. Zhao L. Li R. Liu K. Wang S. (2019). CHCHD2 Promotes Hepatocellular Carcinoma and Indicates Poor Prognosis of Hepatocellular Carcinoma Patients. J. Cancer 10, 6822–6828. 10.7150/jca.31158 31839816 PMC6909951

[B46] Yin X. Xia J. Sun Y. Zhang Z. (2020). CHCHD2 Is a Potential Prognostic Factor for NSCLC and Is Associated with HIF-1a Expression. Bmc Pulm. Med. 20, 40. 10.1186/s12890-020-1079-0 32054470 PMC7020603

[B47] Youle R. J. Narendra D. P. (2011). Mechanisms of Mitophagy. Nat. Rev. Mol. Cel Biol 12, 9–14. 10.1038/nrm3028 PMC4780047 21179058

[B48] Zacksenhaus E. Shrestha M. Liu J. C. Vorobieva I. Chung P. E. D. Ju Y. (2017). Mitochondrial OXPHOS Induced by RB1 Deficiency in Breast Cancer: Implications for Anabolic Metabolism, Stemness, and Metastasis. Trends Cancer 3, 768–779. 10.1016/j.trecan.2017.09.002 29120753

[B49] Zhang Y. Du W. Chen Z. Xiang C. (2017). Upregulation of PD-L1 by SPP1 Mediates Macrophage Polarization and Facilitates Immune Escape in Lung Adenocarcinoma. Exp. Cel Res. 359, 449–457. 10.1016/j.yexcr.2017.08.028 28830685

[B50] Zheng H. Zhou C. Lu X. Liu Q. Liu M. Chen G. (2018). DJ-1 Promotes Survival of Human colon Cancer Cells under Hypoxia by Modulating HIF-1α Expression through the PI3K-AKT Pathway. Cancer Manag. Res. 10, 4615–4629. 10.2147/CMAR.S172008 30410397 PMC6199970

[B51] Zhou W. Ma D. Sun A. X. Tran H.-D. Ma D.-l. Singh B. K. (2019). PD-linked CHCHD2 Mutations Impair CHCHD10 and MICOS Complex Leading to Mitochondria Dysfunction. Hum. Mol. Genet. 28, 1100–1116. 10.1093/hmg/ddy413 30496485 PMC6423417

[B52] Zhu Z. Zhang H. Chen B. Liu X. Zhang S. Zong Z. (2020). PD-L1-Mediated Immunosuppression in Glioblastoma Is Associated with the Infiltration and M2-Polarization of Tumor-Associated Macrophages. Front. Immunol. 11, 588552. 10.3389/fimmu.2020.588552 33329573 PMC7734279

