# Supplementary material for: Significance of Parkinson Family Genes in the Prognosis and Treatment Outcome Prediction for Lung Adenocarcinoma
Source: Front Mol Biosci. 2021 Sep 20;8:735263. doi: 10.3389/fmolb.2021.735263 (PMC8488091; doi:10.3389/fmolb.2021.735263)
Supplement: Supplementary file 1 [file DataSheet1.PDF]

Supplementary Material

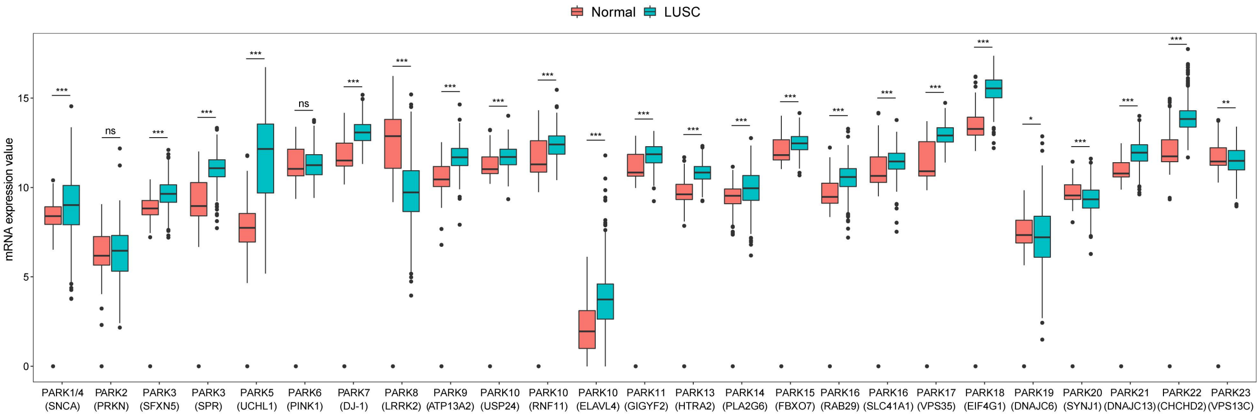

Supplementary Figure S1. Differences in the mRNA expression of 25 Parkinson family genes in LUSC and normal tissues in the TCGA and GTEx databases (normal = 397, LUSC = 498).

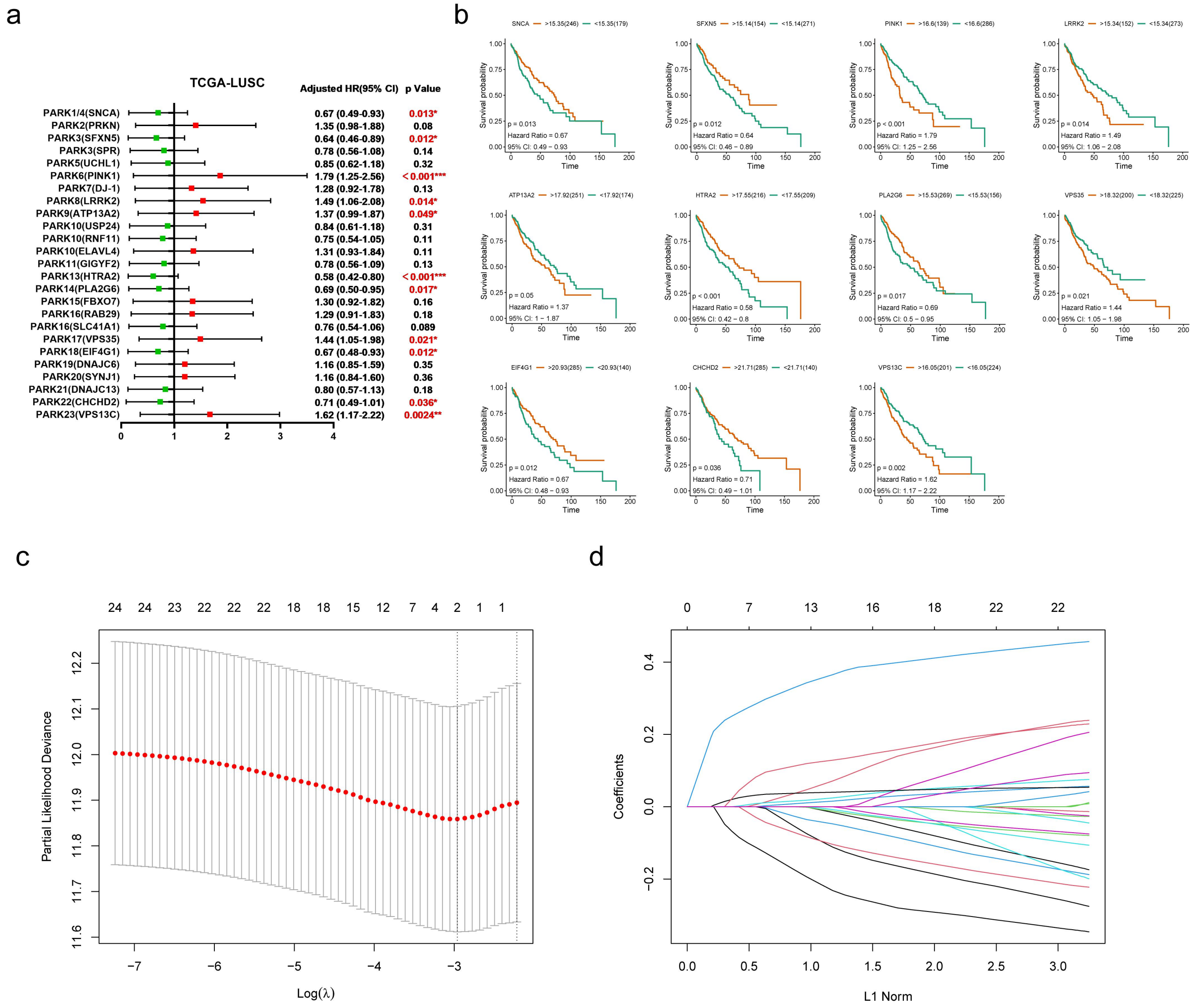

Supplementary Figure S2. The clinical significance of Parkinson family genes for the prognosis of LUSC and the LASSO regression model. (A) LUSC prognostic forest plot of 25 Parkinson family genes from the TCGA database. (B) 11 Parkinson family genes with significant differences based on the prognosis of 423 LUSC patients. (C) and (D) LASSO regression prognostic model for the 423 LUSC patients.

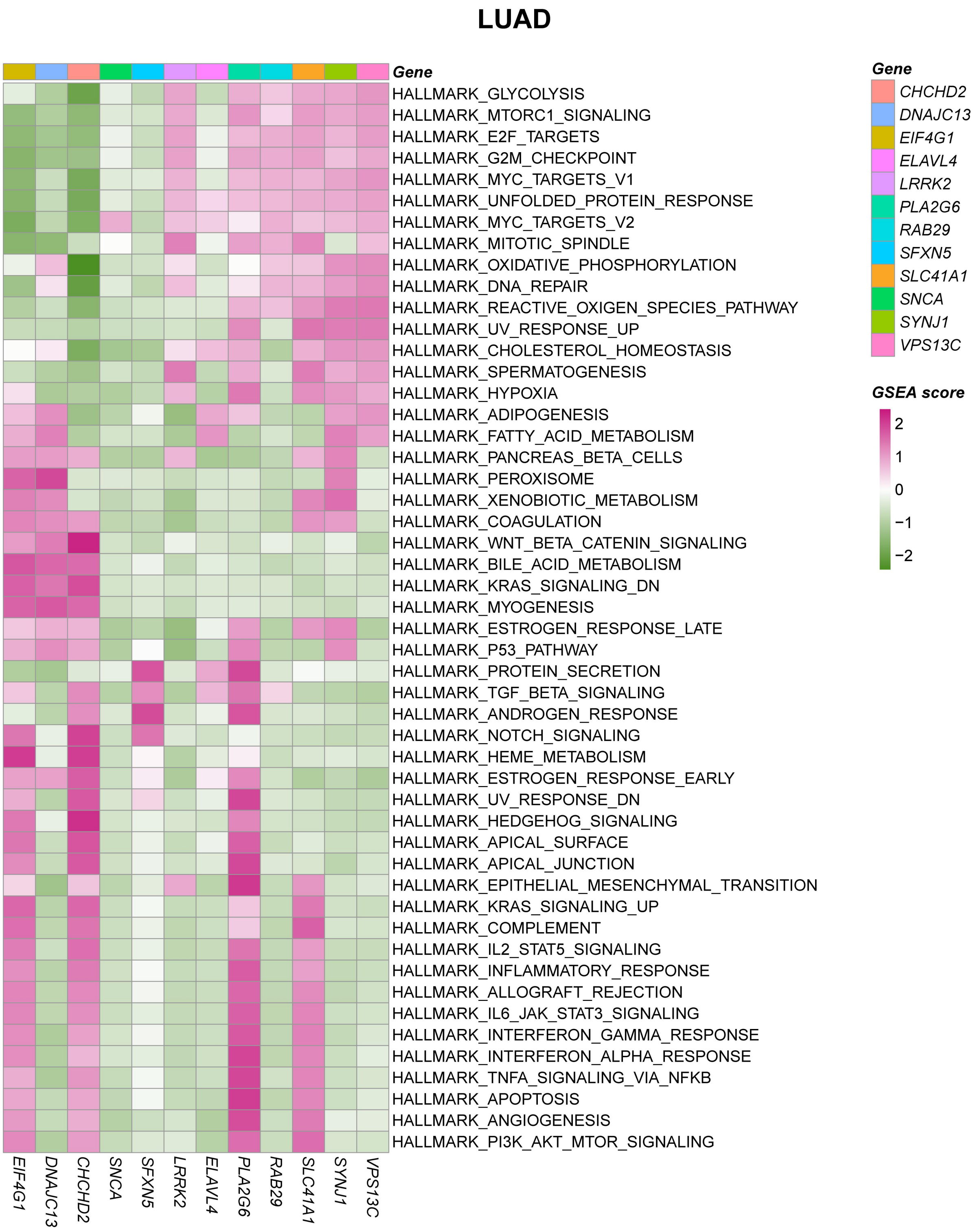

Supplementary Figure S3. Differences in pathway activities scored by GSEA between high and low expression group of 12 prognosis-related Parkinson genes in LUAD. GESA score was scaled by mean-centering and transformed to a scale ranging from -2 to 2, green represents low scores, whereas red denotes high scores.

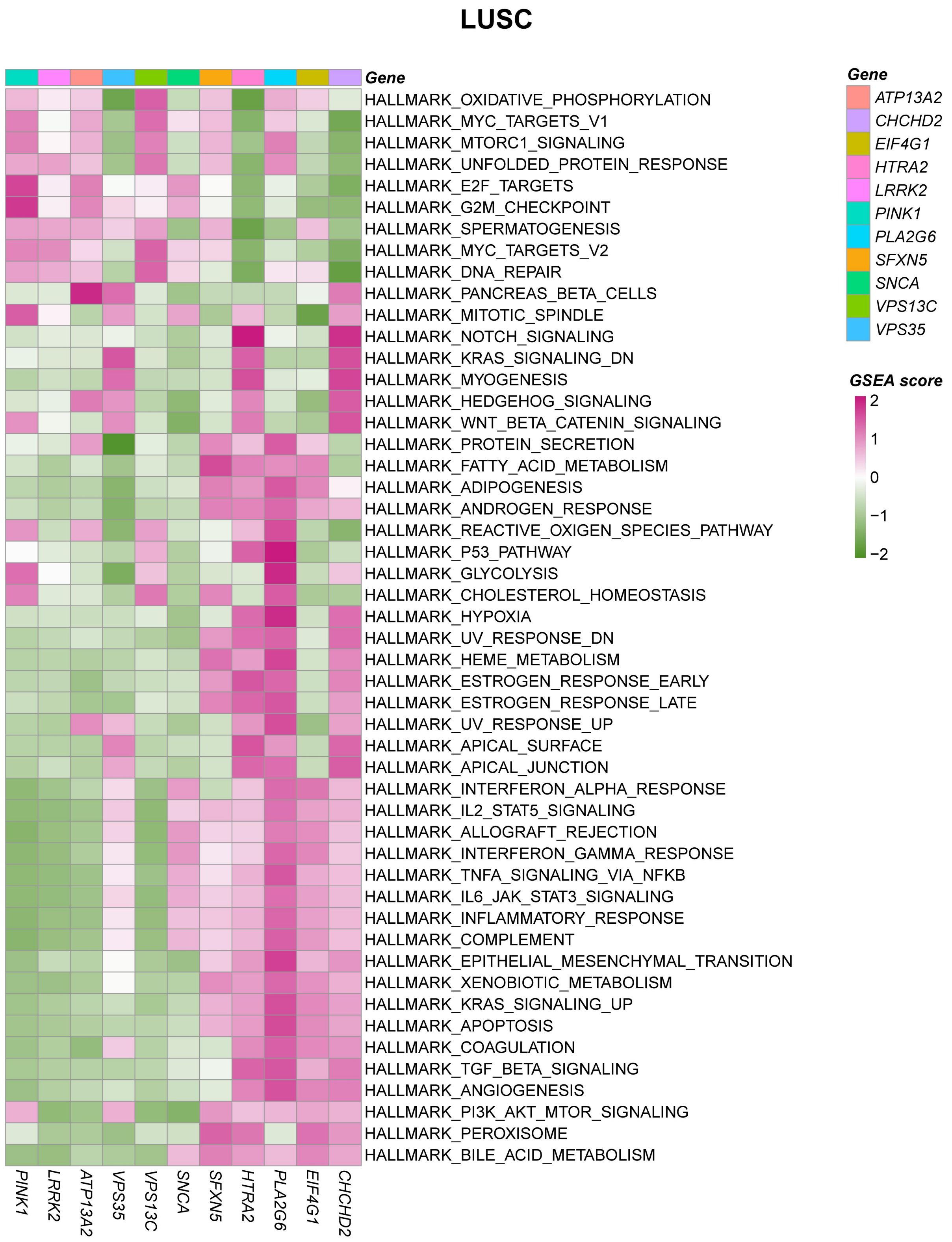

Supplementary Figure S4. Differences in pathway activities scored by GSEA between high and low expression group of 11 prognosis-related Parkinson genes in LUSC. GESA score was scaled by mean-centering and transformed to a scale ranging from -2 to 2, green represents low scores, whereas red denotes high scores.

Correlation analysis of gene expression between 7-gene signature and KEGG map05223 (Non-small cell lung cancer)

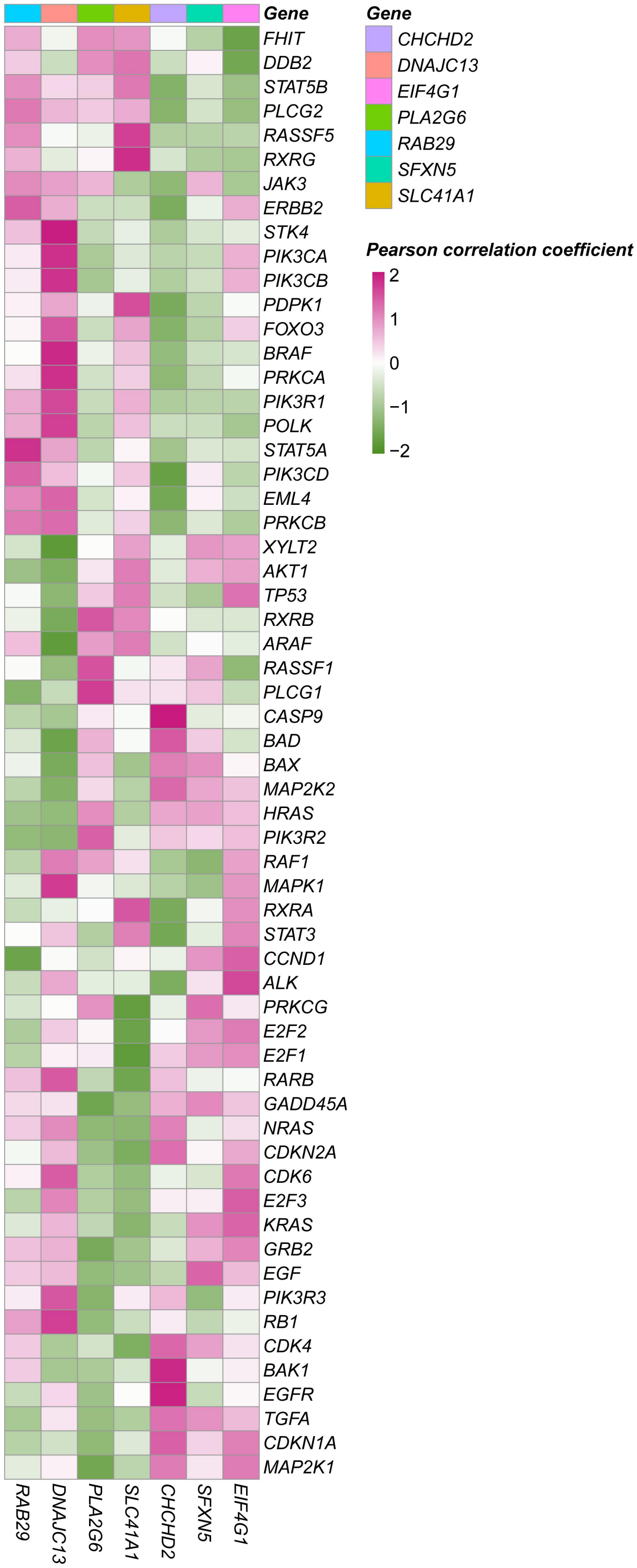

Supplementary Figure S5. The expression correlation between the 7-gene signature of Parkinson genes and cancer progression marker genes from KEGG map05223. Pearson correlation coefficient was scaled by mean-centering and transformed to a scale ranging from -2 to 2, green represents low scores, whereas red denotes high scores.

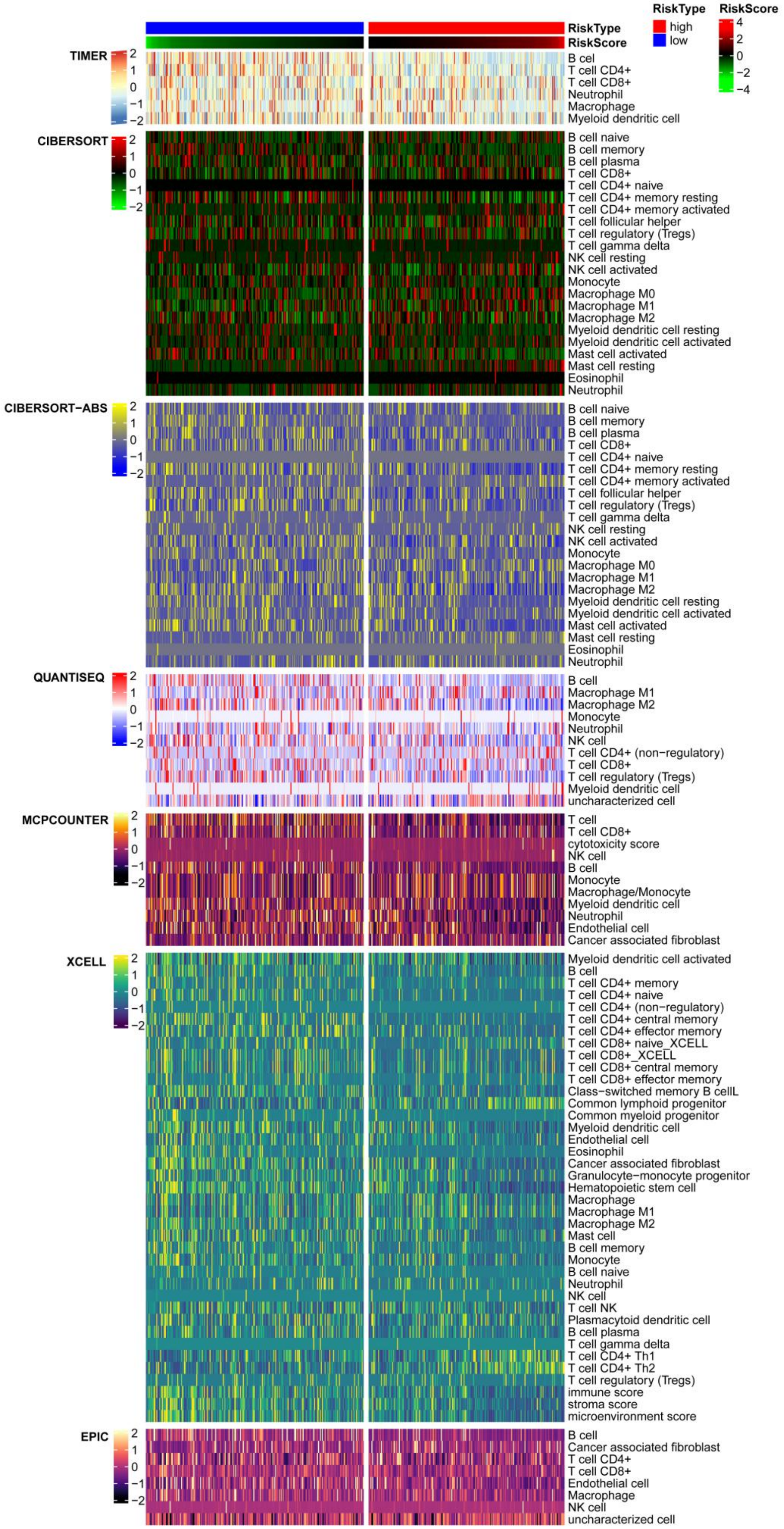
RiskType
RiskScore
high
low
4
2
0
−2
−4
TIMER
B cel
T cell CD4+
T cell CD8+
Neutrophil
Macrophage
Myeloid dendritic cell
CIBERSORT
B cell naive
B cell memory
B cell plasma
T cell CD8+
T cell CD4+ naive
T cell CD4+ memory resting
T cell CD4+ memory activated
T cell follicular helper
T cell regulatory (Tregs)
T cell gamma delta
NK cell resting
NK cell activated
Monocyte
Macrophage M0
Macrophage M1
Macrophage M2
Myeloid dendritic cell resting
Myeloid dendritic cell activated
Mast cell activated
Mast cell resting
Eosinophil
Neutrophil
CIBERSORT−ABS
B cell naive
B cell memory
B cell plasma
T cell CD8+
T cell CD4+ naive
T cell CD4+ memory resting
T cell CD4+ memory activated
T cell follicular helper
T cell regulatory (Tregs)
T cell gamma delta
NK cell resting
NK cell activated
Monocyte
Macrophage M0
Macrophage M1
Macrophage M2
Myeloid dendritic cell resting
Myeloid dendritic cell activated
Mast cell activated
Mast cell resting
Eosinophil
Neutrophil
QUANTISEQ
B cell
Macrophage M1
Macrophage M2
Monocyte
Neutrophil
NK cell
T cell CD4+ (non−regulatory)
T cell CD8+
T cell regulatory (Tregs)
Myeloid dendritic cell
uncharacterized cell
MCPCOUNTER
T cell
T cell CD8+
cytotoxicity score
NK cell
B cell
Monocyte
Macrophage/Monocyte
Myeloid dendritic cell
Neutrophil
Endothelial cell
Cancer associated fibroblast
XCELL
Myeloid dendritic cell activated
B cell
T cell CD4+ memory
T cell CD4+ naive
T cell CD4+ (non−regulatory)
T cell CD4+ central memory
T cell CD4+ effector memory
T cell CD8+ naive_XCELL
T cell CD8+_XCELL
T cell CD8+ central memory
T cell CD8+ effector memory
Class−switched memory B cellL
Common lymphoid progenitor
Common myeloid progenitor
Myeloid dendritic cell
Endothelial cell
Eosinophil
Cancer associated fibroblast
Granulocyte−monocyte progenitor
Hematopoietic stem cell
Macrophage
Macrophage M1
Macrophage M2
Mast cell
B cell memory
Monocyte
B cell naive
Neutrophil
NK cell
T cell NK
Plasmacytoid dendritic cell
B cell plasma
T cell gamma delta
T cell CD4+ Th1
T cell CD4+ Th2
T cell regulatory (Tregs)
immune score
stroma score
microenvironment score
EPIC
B cell
Cancer associated fibroblast
T cell CD4+
T cell CD8+
Endothelial cell
Macrophage
NK cell
uncharacterized cell

Supplementary Figure S6. Seven different methods (TIMER, CIBERSORT, CIBERSORT-ABS, QUANTISEQ, MCPCOUNTER, XCELL, EPIC) for the analysis of differences in immune cell infiltration between high- and low-risk groups in LUAD based on TCGA data.

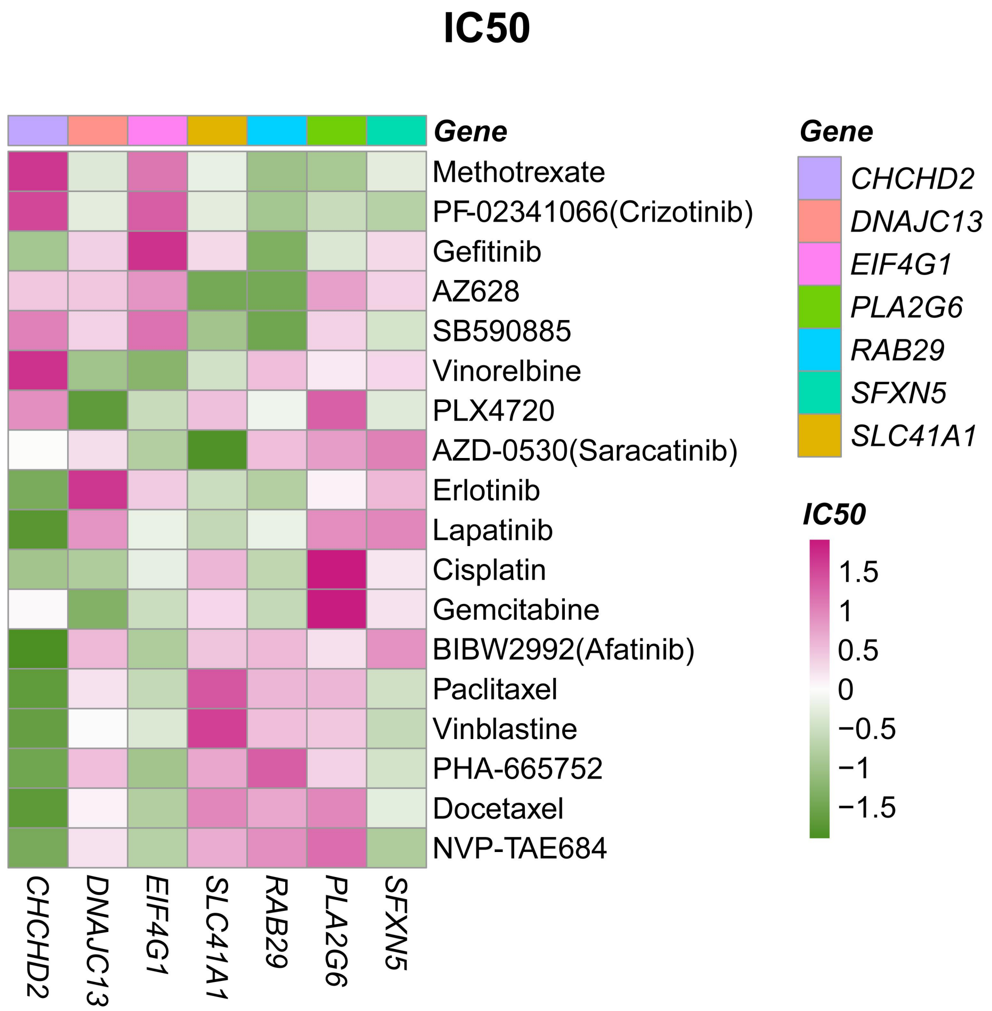

Supplementary Figure S7. Prediction of drug efficacy impact for the Parkinson genes of 7-gene signature to conventional chemotherapy drugs and some targeted drugs for lung cancer in TCGA-LUAD cohort based on GDSC database. IC50 was scaled by mean-centering and transformed to a scale ranging from -1.5 to 1.5, green represents low scores, whereas red denotes high scores.
